# Alpha-Asarone Protects Endothelial Cells from Injury by Angiotensin II

**DOI:** 10.1155/2014/682041

**Published:** 2014-03-18

**Authors:** Hai-Xia Shi, Jiajun Yang, Tao Yang, Yong-Liang Xue, Jun Liu, Ya-Juan Li, Dan-Dan Zhang, Jin-Wen Xu, Ka Bian

**Affiliations:** ^1^Department of Traditional Chinese Medicine, Third People's Hospital Affiliated to Shanghai Jiao Tong University School of Medicine, 280 Mohe Road, Shanghai 201999, China; ^2^Murad Research Center for Modernization Chinese Medicine, Shanghai University of Traditional Chinese Medicine, 1200 Cailun Road, Shanghai 201203, China; ^3^Department of Neurology, Sixth People's Hospital Affiliated to Shanghai Jiao Tong University, 600 Yishan Road, Shanghai 200233, China; ^4^Institute of Liver Diseases, Shuguang Hospital Affiliated to Shanghai University of Traditional Chinese Medicine, Zhangheng Road, Zhangjiang Hi-Tech Park, Shanghai 201210, China; ^5^Department of Biochemistry and Molecular Biology, George Washington University, 2300 I Street, Ross Hall 543, Washington, DC 20037, USA

## Abstract

**α**-Asarone is the major therapeutical constituent of *Acorus tatarinowii* Schott. In this study, the potential protective effects of **α**-asarone against endothelial cell injury induced by angiotensin II were investigated *in vitro*. The EA.hy926 cell line derived from human umbilical vein endothelial cells was pretreated with **α**-asarone (10, 50, 100 µmol/L) for 1 h, followed by coincubation with Ang II (0.1 µmol/L) for 24 h. Intracellular nitric oxide (NO) and reactive oxygen species (ROS) were detected by fluorescent dyes, and phosphorylation of endothelial nitric oxide synthase (eNOS) at Ser^1177^ was determined by Western blotting. **α**-Asarone dose-dependently mitigated the Ang II-induced intracellular NO reduction (*P* < 0.01 versus model) and ROS production (*P* < 0.01 versus model). Furthermore, eNOS phosphorylation (Ser^1177^) by acetylcholine was significantly inhibited by Ang II, while pretreatment for 1 h with **α**-asarone partially prevented this effect (*P* < 0.05 versus model). Additionally, cell viability determined by the MTT [3-(4,5-dimethylthiazol-2-yl)-2,5-diphenyltetrazolium bromide] assay (105~114.5% versus control, *P* > 0.05) was not affected after 24 h of incubation with **α**-asarone at 1–100 µmol/L. Therefore, **α**-asarone protects against Ang II-mediated damage of endothelial cells and may be developed to prevent injury to cardiovascular tissues.

## 1. Introduction

Endothelial cells play key roles in maintaining homeostasis of blood vessels, such as by regulating the vascular tone; inhibiting the proliferation of smooth muscle cells; and preventing inflammation, platelet aggregation, and coagulation [1]. However, reduction in bioavailable nitric oxide (NO) and excess production of reactive oxygen species (ROS) are important contributors to endothelial cell dysfunction, which is the initial factor in development of vascular diseases. It has been suggested that the increase of angiotensin II (Ang II) level in the blood causes endothelial cell dysfunction during the pathogenesis of such conditions as atherosclerosis (AS) and hypertension. The underlying mechanisms involve the following: an increase in permeability of vascular endothelial cells or intima, which contributes to lipid deposition and invasion of harmful substances; activation of NF-*κ*B, thereby promoting the endothelial cells to express monocyte chemotactic protein-1 (MCP-1), soluble intercellular adhesion molecule-1 (sICAM-1), and interleukin-6 (IL-6), which aggravate inflammatory reactions; activation of the reduced form of nicotinamide-adenine dinucleotide (NADH) and reduced nicotinamide adenine dinucleotide phosphate (NADPH) oxidase of the cell membrane, leading to oxidative stress; and expression of vasoactive substances and even apoptosis of endothelial cells [2]. Additionally, a large number of studies have shown that Ang II induces endothelial nitric oxide synthase (eNOS) uncoupling through the activation of NADPH oxidase, reducing the content of BH_4_, and contributes to the reduction of NO, eventually resulting in increased ROS and endothelial cell dysfunction [3]. Ang II at 0.1 *μ*mol/L has been shown to inhibit the phosphorylation of eNOS at Ser^1177^ and the production of NO stimulated by insulin in human vascular endothelial cells (HUVECs) [4]. Furthermore, Ang II can also upregulate the phosphorylation of eNOS at Tyr^657^, thereby inhibiting its activity, decreasing the production of NO, and mediating functional damage of endothelial cells [5].

As the principle active component of the medicinal plant of* Acorus tatarinouii* Schott, *α*-asarone (2,4,5-trimethoxy-1-propenyl benzene) exhibits pharmacological activities that mirror those of the whole plant extract. It harbors antiasthma [6], antiepilepsy [7], and cardiovascular protective [8, 9] properties, as well as the ability to lower lipemia and cholesteremia [10]. Recent studies have attributed the neuroprotective [11, 12] and antiepileptic [13] effects of *α*-asarone to its antioxidant properties [14]. Despite reports of its hypolipidemic [15, 16] and antiplatelet [17] effects, the potential protective activities of *α*-asarone on vascular endothelial cells have not yet been determined.

Our previous study showed that *α*-asarone possessed a endothelium-dependent vasodilation property, and in normal HUVEC-derived EA.hy926 cells, *α*-asarone has been shown to upregulate the expression of phosphorylated eNOS (Ser^1177^), causing an increase in the production of NO. In the present study, the protective effects of *α*-asarone against endothelial dysfunction induced by Ang II and the underlying molecular mechanisms were investigated. The results showed effective bioactivities of *α*-asarone in improving the Ang II-mediated reduction in NO and ROS production in endothelial cells in a dose-dependent manner. Furthermore, the inhibition of acetylcholine (ACh)-mediated phosphorylation of eNOS Ser^1177^ induced by Ang II could be partially reversed by pretreatment with *α*-asarone at doses which did not influence cellular proliferation.

## 2. Materials and Methods

### 2.1. Chemicals

All chemicals used in this study were purchased from Sigma (St Louis, MO, USA) unless otherwise specified. The stock solution of *α*-asarone (10^5^ *μ*mol/L) was dissolved in dimethyl sulfoxide (DMSO) and then diluted in culture medium to a working concentration. Ang II and ACh were prepared in distilled water.

### 2.2. Cells

The EA.hy926 cell line was purchased from American Type Culture Collection (ATCC, Manassas, VA, USA, CRL-2922) and cultured in 10 cm diameter Petri dishes with Dulbecco's Modified Eagle Medium (DMEM) containing 10% fetal calf serum (FCS) and supplemented with 1% hypoxanthine-aminopterin-thymidine based on the manufacturer's recommendations. The cells were incubated at 37°C in humidified air containing 5% CO_2_. The 4th to 8th generations of cells were used in the experiments.

### 2.3. Determining Effects of Ang II on Intracellular ROS/NO Levels

EA.hy926 cells were subcultured at 50,000/well in 96-well plates. Before the experiment, the medium was changed to serum-free and phenol red-free RPMI 1640 medium and cultured overnight (8~10 h) at 37°C with 5% CO_2_. The model group was cultured with phenol red-free RPMI 1640 medium containing Ang II at a final concentration of 0.1 *μ*mol/L, while the control group was cultured with the same medium without Ang II. Both groups were cultivated for 24 h. As a positive control, the NO synthesis inhibitor L-NG-nitro arginine methyl ester (L-NAME) at the final concentration of 100 *μ*mol/L was used to treat EA.hy926 cells for 1 h before detection of NO and ROS levels. Dihydroethidium (DHE, final concentration 2 *μ*mol/L) and difluorofluorescein diacetate (DAF-FM DA, final concentration 5 *μ*mol/L) diluted with serum-free and phenol red-free RPMI1640 medium were loaded into the cells by incubation in the dark for 30 min at 37°C before detection. The supernatant was discarded, and the cells were washed with phosphate buffer saline (PBS) 2-3 times before adding 200 *μ*L of PBS for fluorescence detection using a full wavelength multifunctional microplate reader. For NO detection, ACh was added at the final concentration of 10 *μ*mol/L and incubated for 10~30 min before detection.

### 2.4. Determining Protective Effect of *α*-Asarone against Ang II-Mediated Injury of Endothelial Cells by Measuring ROS/NO Content

EA.hy926 cells were subcultured at 50,000/well in 96-well plates as in the experiment above and pretreated with *α*-asarone at different concentrations (10, 50, and 100 *μ*mol/L) for 1 h. Subsequently, Ang II (0.1 *μ*mol/L) was added to the cultures and incubated for 24 h at 37°C. Thereafter, the cells were loaded with the fluorescent probes for 30 min, and then the extracellular residual dyes were washed away before detection as described above.

### 2.5. Determining Protective Effects of *α*-Asarone against Ang II-Mediated Injury of Endothelial Cells by Detection of ACh-Induced eNOS Ser^1177^ Phosphorylation

EA.hy926 cells were seeded in 10 cm Petri dishes at 1 × 10^6^/dish. When the cells had grown to 90% confluency, they were preincubated in serum-free DMEM with *α*-asarone (10 *μ*mol/L) for 1 h and then treated with Ang II at 0.1 *μ*mol/L for 24 h. The control group was incubated in serum-free DMEM. After the incubation, the medium of the treated group was switched to DMEM with ACh at 10 *μ*mol/L, and then cells were incubated for 0, 2, 5, 10, 15, 30, and 60 min. Total cellular proteins were extracted for Western blot analysis of total eNOS and phosphorylated eNOS (Ser^1177^). The cell culture dish was placed in ice and the cells was washed with ice-cold PBS; after aspirating the PBS, add ice-cold lysis buffer and scrape adherent cells off the dish using a cold plastic cell scraper; then, gently transfer the cell suspension into a precooled microcentrifuge tube. After that, maintain constant agitation for 30 minutes at 4°C, spin at 16,000 ×g for 20 minutes in a 4°C precooled centrifuge, transfer the supernatant to a fresh tube kept on ice, and discard the pellet. Remove a small volume (20 *μ*L) of lysate to perform a protein assay and add an equal volume of 2x Laemmli sample buffer. Then load and run the gel from 1 to 2 hours at 100 V, before transferring the protein from the gel to the membrane; after that, block the membrane for 1 hour at room temperature using 5% blocking solution and incubate membrane with appropriate dilutions of primary antibody in 5% blocking solution overnight at 4°C. Then, wash the membrane in three washes of TBST, 5 minutes each. Incubate the membrane with the recommended dilution of labeled secondary antibody in 5% blocking buffer in TBST at room temperature for 1 hour. Then, wash the membrane in three washes of TBST, 5 minutes each. To acquire images, use darkroom development techniques for chemiluminescence. Quantity 1 software (Bio-Rad Laboratories, Inc. USA) was used for immunoblot analysis.

### 2.6. MTT [3-(4,5-Dimethylthiazol-2-yl)-2,5-diphenyltetrazolium bromide] Cell Viability Assay for Determination of Drug Safety

EA.hy926 cells were subcultured at 5,000/well in 96-well plates and switched to serum-free DMEM overnight (8~10 h) before the experiment when *α*-asarone was added at different concentrations (1, 5, 10, 50, and 100 *μ*mol/L) and incubated for 24 h. The cell viability assay was conducted by adding 20 *μ*L of working MTT solution per well and incubating for 4 h at 37°C in 5% CO_2_. After discarding the supernatant, 150 *μ*L of DMSO was added per well, mixed in a microplate reader, and reacted for 10 min at room temperature. The absorbance was detected at 570 nm to calculate the cell viability.

### 2.7. Statistical Analysis

Values were expressed as means ± standard deviation (SD). Statistical significance of results between different groups was calculated using one-way analysis of variance (ANOVA). All data analyses were performed with SPSS v15.0 (SPSS, Chicago, USA). A *P* value of <0.05 was considered significant.

## 3. Results

### 3.1. Ang II Increases ROS and Decreases NO in EA.hy926 Cells

**Figure 1:**
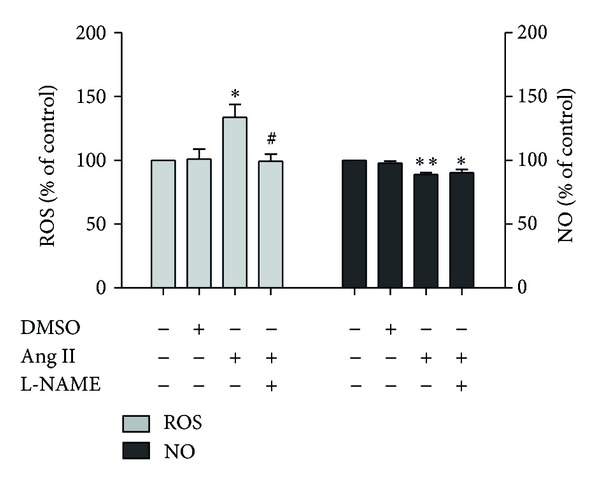
ROS/NO levels in EA.hy926 cells induced by Ang II. Cells were treated with Ang II at 0.1 *μ*mol/L for 24 h. The intracellular ROS and NO levels were detected with the permeable fluorescent dyes DHE and DAF-FM DA, respectively. DMSO was used as the solvent control at the concentration of 1%. L-NAME (100 *μ*mol/L) was used to sustain the changes of ROS and NO related to eNOS. Data are expressed as means ± SD from three independent experiments. *P < 0.05 versus control; **P < 0.01 versus control; ^#^ *P* < 0.05 versus model.

As shown in Figure 1, after incubation with Ang II (0.1 *μ*mol/L) for 24 h, the intracellular ROS level statistically and significantly increased by 34% when compared with control group (*P* < 0.05). In the group pretreated with the NO synthesis inhibitor L-NAME before exposure to Ang II, the ROS level was decreased (*P* < 0.05) compared with the model (Ang II only) group, suggesting that the elevation of ROS by Ang II was due to eNOS uncoupling. Meanwhile, the NO level in the model group decreased by 11% when compared with control group, and this difference was statistically significant (*P* < 0.01). These results indicated that the model was constructed successfully (Figure 1).

### 3.2. *α*-Asarone Protects against Injury to Endothelial Cells by Attenuating Ang II-Mediated Changes in Intracellular ROS/NO

**Figure 2:**
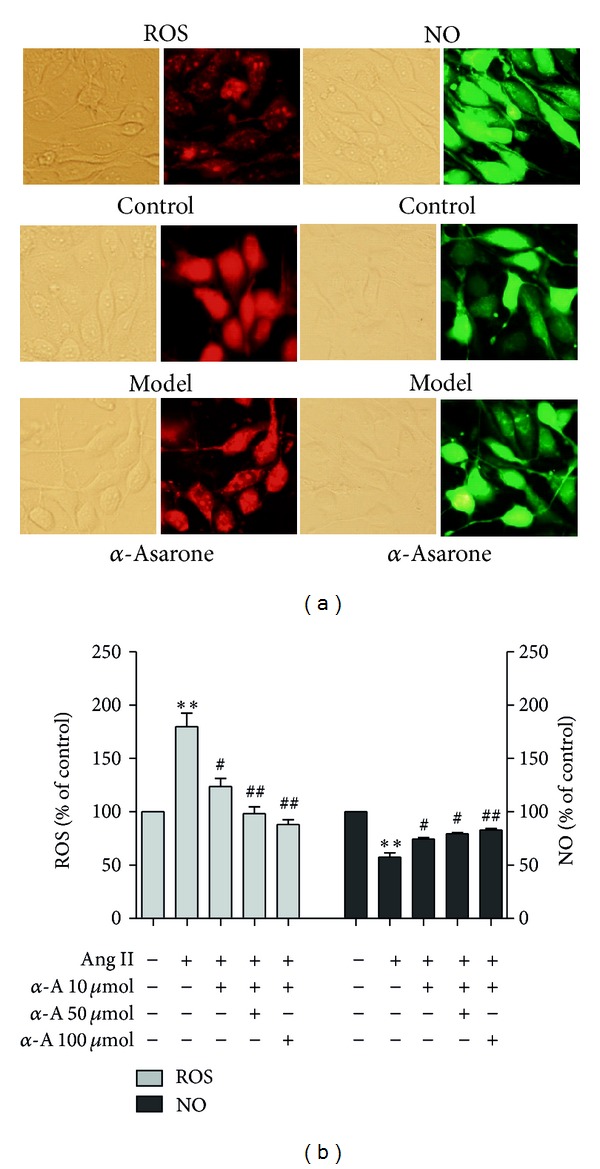
Protection of *α*-asarone against changes in ROS/NO levels in EA.hy926 cells induced by Ang II. Cells were treated with serum-free and phenol red-free RPMI 1640 medium (Control group) or Ang II (Model group) or pretreated with 10, 50 and 100 *μ*mol/L of *α*-asarone (*α*-A) for 1 h, followed by Ang II at 0.1 *μ*mol/L for 24 h. The intracellular ROS and NO levels were detected with fluorescent dyes DHE and DAF-FM DA, respectively. Magnification, ×40 (a). Data are expressed as means ± SD from three independent experiments. **P < 0.01 versus control; ^#^ *P* < 0.05 versus model; ^##^ *P* < 0.01 versus model (b).

Pretreatment of EA.hy926 cells with *α*-asarone at concentrations of 10, 50, or 100 *μ*mol/L could significantly inhibit the intracellular ROS levels induced by Ang II, while at the same time it also minimized the decrease of intracellular NO levels induced by Ang II (Figure 2). These results suggested that *α*-asarone may have protective effects against oxidative stress injury of endothelial cells induced by Ang II.

### 3.3. *α*-Asarone Reverses Ang II-Mediated Inhibition of ACh-Induced Phosphorylation of eNOS (Ser^1177^) 

**Figure 3:**
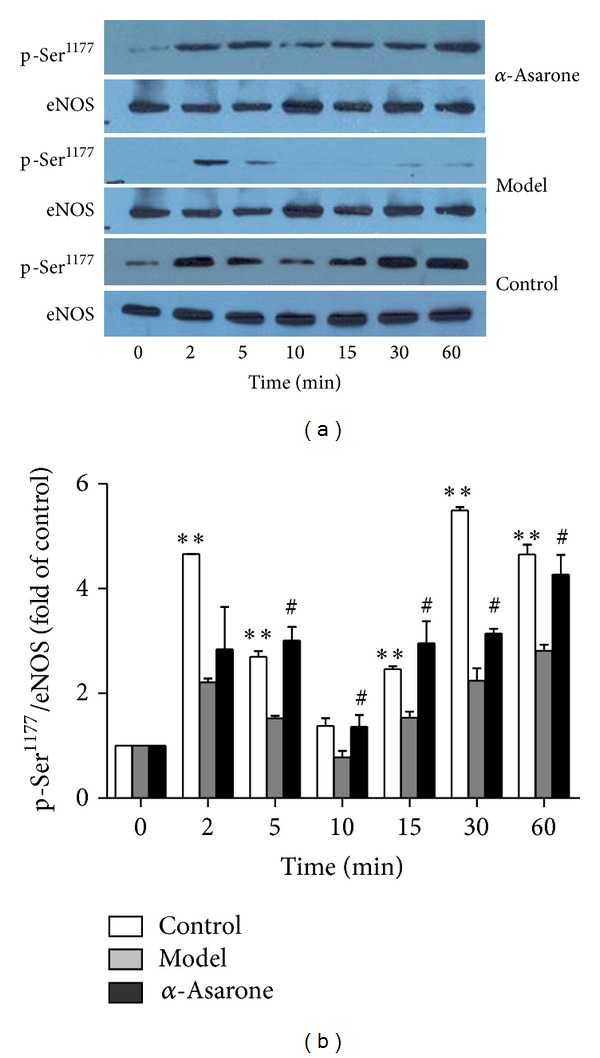
Protection of *α*-asarone against Ang II-mediated impairment of the ACh- induced eNOS Ser^1177^ phosphorylation in EA.hy926 cells. Cells were stimulated either with ACh (10 *μ*mol/L) only (a, Control), incubated with Ang II (0.1 *μ*mol/L) for 24 h, followed by stimulation with ACh (a, Model), or pr-treated with *α*-asarone (10 *μ*mol/L) for 1 h and then incubated with Ang II (0.1 *μ*mol/L) for 24 h, followed by stimulation with ACh (a, *α*-asarone) for the indicated times. Cells were lysed and subjected to Western blot analysis of phosphorylated eNOS at Ser^1177^ or for total eNOS protein. Data are expressed as means ± SD from three independent experiments. *P < 0.05; **P < 0.01 versus 0 min group; ^#^ *P* < 0.05 versus model group (b).

The ability of the eNOS agonist ACh (10 *μ*mol/L) to upregulate phosphorylation of eNOS at the Ser^1177^ site was confirmed, the effect of which was exhibited as twin peaks (Figure 3, control). The level of eNOS Ser^1177^ phosphorylation increased significantly after 2 min of ACh treatment and then dropped sharply; thereafter, it increased slowly, reaching the second peak at 30 min and achieving a level which was 5.5 times higher than that of the control group (0 min) (*P* < 0.01) (Figure 3, Control).

Meanwhile, treatment of the EA.hy926 cells with Ang II (0.1 *μ*mol/L) for 24 h before stimulation with ACh (10 *μ*mol/L) for 0–60 min had a profound effect on the ACh-mediated induction of eNOS Ser^1177^ phosphorylation (Figure 3, Model). While there was no significant change in total eNOS protein, the eNOS Ser^1177^ phosphorylated protein level initially induced by ACh was minimal, disappearing over time, and the difference was statistically significant compared with the control group. However, when the EA.hy926 cells were pretreated with *α*-asarone (10 *μ*mol/L) for 1 h, followed by incubation with Ang II (0.1 *μ*mol/L) for 24 h and stimulation with ACh (10 *μ*mol/L) for 0–60 min, the level of phosphorylated eNOS (Ser^1177^) protein recovered to some extent over time and was close to that of the control group but significantly different in comparison with the model group (*P* < 0.05) (Figure 3, *α*-asarone).

### 3.4. *α*-Asarone at Concentrations up to 100 *μ*mol/L Does Not Affect Proliferation of EA.hy926 Cells

**Figure 4:**
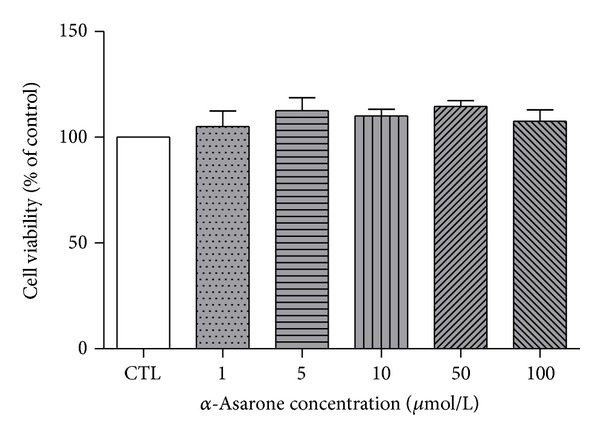
Viability of EA.hy926 cells treated with *α*-asarone. Cells were treated with various concentrations of *α*-asarone (1–100 *μ*mol/L) for 24 h, and then cell viability was determined by the MTT assay. There was no significant difference between control (CTL) and *α*-asarone treated groups. Data are expressed as means ± SD from three independent experiments.

The effect of *α*-asarone on proliferation of EA.hy926 cells was determined using the MTT method. After incubation with different concentrations of *α*-asarone (1~100 *μ*mol/L) for 24 h, no significant difference in the proliferative capacity of EA.hy926 cells was observed among *α*-asarone-treated and control groups (Figure 4). This result demonstrated that treatment with *α*-asarone in this concentration range for 24 h had no effect on growth of EA.hy926 cells.

## 4. Discussion

The dysfunction of endothelial cells is the initiating factor and pathological basis of vascular lesions. As an important mediator of endothelial cell function, NO plays a key role in the maintenance of vascular homeostasis, and eNOS is the main enzyme driving the synthesis of endothelium-derived NO [18]. As eNOS is a constitutively expressed enzyme, its activity and function are mainly regulated by posttranslational modifications, acetylation, intracellular calcium concentration, phosphorylation, and S-nitrosylation. Various protein families and signaling pathways, including calmodulin (CaM), caveolin-1, slow bradykinin B2 receptors, heat shock protein 90 (hsp90), and protein kinase B (PKB/Akt), are involved in the regulation of eNOS subcellular localization, catalytic function, and biological activity. The present study focused on the phosphorylation of eNOS as the primary posttranslational modification regulating its activity. The critical regulatory sites include Ser^1177^, Ser^635^, Ser^617^, Thr^495^, and Ser^116^, with eNOS activity being increased by phosphorylation at the former three sites and decreased by phosphorylation at the latter two sites [19]. In pathological conditions, the imbalance of the regulatory network will lead to the decrease of eNOS activity and consequently the decrease in NO production.

In a previous investigation in cultured bovine aortic endothelial cells, Ang II was found to increase H_2_O_2_ by activation of NADPH oxidase, causing the downregulation of intracellular dihydrofolate reductase (DHFR), decrease in BH_4_ levels, and uncoupling of eNOS, and ultimately leading to excess intracellular ROS and endothelial cell dysfunction [3]. In addition, Ang II and H_2_O_2_-mediated eNOS Tyr^657^ phosphorylation have been shown to inhibit eNOS activity and reduce NO production [5]. Ang II can also inhibit insulin-stimulated eNOS Ser^1177^ phosphorylation and NO production, which may lead to the dysfunction of endothelial cells [4]. Furthermore, inhibition of large conductance calcium-activated potassium channels (BKCa) is one of the early responses, involving protein G, to Ang II-mediated damage to vascular endothelial cells [19]. Ginkgo biloba extract, which can activate BKCa and defend against the adverse effects of Ang II on BKCa, has demonstrated protective effects on endothelial cells. In animal experiments, angiotensin receptor antagonists were confirmed to inhibit rat endothelial cell proliferation, migration, and matrix increase, and may improve endothelial cell function [20].

In this study, *α*-asarone was investigated for its potential effect to preserve endothelial cells against Ang II-mediated damage. The results collectively suggest that *α*-asarone dose-dependently inhibited the Ang II-induced intracellular ROS with an *I*_max⁡_ of 50.60 ± 5.23%. Meanwhile, the intracellular NO level was elevated with an *E*_max⁡_ of 33.24 ± 13.55%. These results indicate that *α*-asarone may improve Ang II-mediated eNOS uncoupling and provide antioxidant protection for endothelial cell function.

In addition, it has been reported that by inhibiting the phosphorylation of eNOS Ser^1177^, Ang II leads to the downregulation of eNOS activity and NO production, thereby causing endothelial cell dysfunction [4]. In the present study, a model of endothelial cell damage by Ang II was established via modulation of ACh-induced eNOS phosphorylation at Ser^1177^, and the protective effect of *α*-asarone was investigated. In EA.hy926 endothelial cells stimulated with ACh (10 *μ*mol/L) for different times (0–60 min), the phosphorylation of eNOS at Ser^1177^ was increased in a bimodal fashion. With the application of Ang II (0.1 *μ*mol/L) to EA.hy926 cells for 24 h before treatment with ACh (10 *μ*mol/L), eNOS Ser^1177^ phosphorylation disappeared over time, while total eNOS protein levels were unchanged. In order to investigate whether *α*-asarone protected against Ang II-induced injury via eNOS Ser^1177^ phosphorylation, EA.hy926 cells were pretreated with *α*-asarone (10 *μ*mol/L) for 1 h before the 24 h incubation with Ang II and subsequent 1 h stimulation with ACh. Over a time course, the expression of phosphorylated eNOS (Ser^1177^) recovered to a level which was close to that of the normal control group and significantly different compared with the model group. Drugs such as ACh classically mediated endothelium-dependent vasodilation by a mechanism of action that involves multiple intracellular signal transduction pathways, that is, (a) cholinergic receptors (mAChR) → G protein-coupled receptor (GPCR) → phospholipase C (PLC)-protein kinase A (PKA) → eNOS phosphorylation; (b) GPCRs → Tyr K (Tyrosine Kinase, Tyr K) → PI3K/Akt → eNOS phosphorylation; and (c) GPCRs → IR (insulin receptor, IR) → IP 3K/Akt → eNOS phosphorylation. As observed in the current study, ACh treatment induced eNOS Ser^1177^ phosphorylation in EA.hy926 endothelial cells within a short period of time (1 h). Therefore, ACh was an efficient tool for examining the efficacy of *α*-asarone against Ang II-mediated inhibition of eNOS activation. Incubation of the EA.hy926 cells with Ang II for 24 h significantly lowered the ACh-induced eNOS Ser^1177^ phosphorylation, suggesting that Ang II may exert endothelial cell damage through this pathway. Meanwhile, pretreatment with *α*-asarone obviously improved the Ang II-mediated inhibition of eNOS Ser^1177^ phosphorylation by ACh, suggesting that *α*-asarone may play a role in protecting endothelial cell function through preserving the pathway to activation of eNOS. Whether *α*-asarone acts synergistically with ACh or by other means to regulate eNOS activity, as the mechanism of protection against Ang II-induced endothelial cell dysfunction, remains to be further explored.

As it has been previously reported in animal experiments and cultured liver cells that long-term treatment with high concentrations of *α*-asarone results in toxicity to liver and reproductive tissues [21–23], the MTT assay was used here to test the effect of a 24 h incubation with *α*-asarone (1–100 *μ*mol/L) on proliferation of EA.hy926 cells. The proliferation of EA.hy926 cells treated with *α*-asarone was not significantly different from that of the control, suggested that concentrations up to 100 *μ*mol/L of *α*-asarone are safe for these cells.

Although *α*-asarone protected endothelial cells by restored eNOS ser^1177^ phosphorylation, the mechanism remains to be further studied. Tissue factor (TF) and plasminogen activator inhibitor-1 (PAI-1) were markers of the endothelial cells injury [24]. It was reported that Ang II induced the expression of tissue TF and PAI-1 in vascular endothelial cells [25]. Thus, we will further investigate the effects of *α*-asarone on TF or PAI-1 in the future.

A previous study reported that *α*-asarone inhibited lipid synthesis and secretion in long-term cultures of adult rat hepatocytes [15]. The neuroprotective effect of *α*-asarone on spatial memory and nitric oxide levels in rats was investigated in recent study [12]. The endothelium-dependent vasodilatory effect of *α*-asarone was studied in this study and we found that *α*-asarone could protect EA.hy926 cells against Ang II-induced injury. However, it is unclear whether *α*-asarone only acts on endothelial cells. We will use different endothelial cell lines to further clarify the effects of *α*-asarone in future studies.

## 5. Conclusion

This study showed that *α*-asarone could protect EA.hy926 cells against injury induced by Ang II, possibly attributed to its antioxidant activity and by modulating eNOS phosphorylation. Further studies are clearly needed to reveal the regulatory effect of *α*-asarone on the other phosphorylated sites in Ang II-induced endothelial cells.
